# A framework of interpretable match results prediction in football with FIFA ratings and team formation

**DOI:** 10.1371/journal.pone.0284318

**Published:** 2023-04-13

**Authors:** Calvin C. K. Yeung, Rory Bunker, Keisuke Fujii

**Affiliations:** 1 Graduate School of Informatics, Nagoya University, Nagoya, Aichi, Japan; 2 RIKEN Center for Advanced Intelligence Project, Fukuoka, Fukuoka, Japan; 3 PRESTO, Japan Science and Technology Agency, Kawaguchi, Saitama, Japan; XJTLU: Xi’an Jiaotong-Liverpool University, CHINA

## Abstract

While forecasting football match results has long been a popular topic, a practical model for football participants, such as coaches and players, has not been considered in great detail. In this study, we propose a generalized and interpretable machine learning model framework that only requires coaches’ decisions and player quality features for forecasting. By further allowing the model to embed historical match statistics, features that consist of significant information, during the training process the model was practical and achieved both high performance and interpretability. Using five years of data (over 1,700 matches) from the English Premier League, our results show that our model was able to achieve high performance with an F1-score of 0.47, compared to the baseline betting odds prediction, which had an F1-score of 0.39. Moreover, our framework allows football teams to adapt for tactical decision-making, strength and weakness identification, formation and player selection, and transfer target validation. The framework in this study would have proven the feasibility of building a practical match result forecast framework and may serve to inspire future studies.

## Introduction

Forecasting match results is a popular subject in football (Also known as “Association Football”, or “Soccer”). Such models are usually applied to model the randomness of a football match as a statistical challenge, or to profit and arbitrage from football betting. With the advancement of machine learning and open-source football data becoming increasingly available, developing models that forecast match results and provide practical utility to bookmakers and bettors, and especially to teams, coaches, and performance analysts, is now more feasible than ever.

Historically, to forecasting football match results, researchers have attempted to fit a distribution to the number of goals a team will score in a football match. In one of the seminal papers in football forecasting, Maher [1] applied an independent Poisson distribution and was able to derive the attacking and defensive rating of a team from the model. Another foundational paper, that by Dixon and Coles [2], built on the basic model structure of [1], modifying it to allow for both incomplete data and data from different divisions, as well as allowing for fluctuations in team performance over time. Apart from the independent Poisson distribution, there have been successful attempts to fit the goal distribution with dependent Poisson, negative binomial, or generalized extreme value distributions [3–6]. Other studies [7–11] have also adopted the attacking and defensive rating based approach. Recently, with the development of machine learning models, more complex models with better performance have been proposed.

During the past decade, researchers began to apply machine learning models such as naive Bayes, tree models, regression, and support vector machines (SVMs). Most researchers used historical match statistics and incorporated alternative features, e.g., Twitter posts [12], weather conditions [13], and human experts’ opinions [14]) as input features. Currently, boosted tree methods (e.g., XGBoost) have achieved the best performance [15–20], and for readers who are interested in prediction accuracy and other measurements in the previous works [21, 22]. As a result, feature engineering is considered to be a more feasible research direction for further performance improvement.

Wheatcroft [23] recently proposed an effective method, generalized attacking performance (GAP) ratings, for feature engineering. In general, the GAP ratings model predicts future, non-rare match statistics using historical match statistics. Non-rare match statistics (e.g., total shots attempted, shots on and off target, and crosses) are statistics regarding important actions that occur more than a few times in a match. They are used since rare match statistics generally provide insignificant information to football match result forecasting models [23]. The future, non-rare match statistics predicted by GAP ratings are able to provide more information (in terms of the Akaike information criterion (AIC)) to a football match result forecasting model than substituting the future, non-rare match statistics with the historical average of the non-rare match statistics [23].

Even though the GAP ratings model is an effective method to perform feature engineering, the GAP ratings features required, the historical match statistics, are features that are based on historical results, meaning that the features are based on past match results. Other common historical result based features include team historical rankings and team ratings (e.g., Elo ratings [24] and Pi ratings [10]). The use of historical result based features limits a model’s practicality to sports participants like coaches and players.

Extending on the practicality aspect, the aim of applying data analysis for a sports team is to assist in winning matches. Some of the most practical ways in which coaches and players in football can improve are by changing player formation and improving player quality. Therefore, a practical model should be interpretable, and able to infer the relationships between formations, players, player qualities, and match results. Further, the model needs to be generalized, as after the team has improved or changed, it is likely that the performance is unseen, however, a generalized model will still be able to infer the relationship of unseen formations, players, and player qualities with match results.

The historical results based features are not practical to sports participants for two reasons. First, when such features are used as input features, it may limit the generalization of the model. Since these features require the match event to have already actually occurred, it is not possible to obtain such data for a changed or improved team beforehand, for example, for team with young players on debut, newly transferred players, or those with new formations. Second, such features may sometimes limit the interpretability of the model. Since historical data are fixed, even if we identify how the historical data affect the future match result, it does not give a direct and clear indication to the team on how they can improve, i.e., the team has no control over historical events. Other non-historical and non-team-related features like expert opinions, weather, and betting odds are also not controllable by the team. However, the difference is that historical based features, typically historical match statistics, are being frequently adopted by studies [10, 23, 25–29], indicating that such features are able to provide a significant amount of information consistently.

To address these issues, we propose a generalized and interpretable football match result forecasting approach. We first solve the problem of lack of control by sports participants by using selected players, formations, and player qualities as features. These features can be directly controlled by sports participants, representing the decisions of coaches and quality of players. Further, we solve the generalization limitation using historical features by applying the concept of non-rare match statistics prediction [23] to embed the match statistics data into our model during training. Thus, the model utilizes the historical match statistics data, but does not require such data, observed over a long period, as input during the deployment of the model.

Our approach can be summarized in two main steps: non-rare match statistics prediction, inspired by [23], and forecasting football match results. In the first step we use a linear regression model to predict the non-rare match statistics, shots and crosses, with the selected formations, players, and player qualities in the same match. This allows our model to consider the influence of coaches’ decisions and player qualities on the non rare match statistics. We should also point out that our method of predicting non-rare match statistics has nothing in common with [23], except that we both aim to predict the non-rare match statistics.

In the second step, forecasting football match results, we concatenate the predicted non-rare match statistics and competition rounds as input features and apply XGBoost to forecast the match result. Consequently, the relationship between the non-rare match statistics and the match result can be revealed. The performances of the approach were then compared with predictions based on betting odds and baseline models. We also discuss the generalization and interpretability of the proposed approaches, as well as how teams can utilize them.

The main contributions of this study are as follows. First, we propose an accurate (compared to predictions based on betting odds) and practical method for football participants in which they can adjust based on coaches’ decisions and player qualities using the information from the model. Such practicality has not been emphasized in previous studies. Second, we provide a new direction for applying historical based features, where such features can be embedded into the model in order to obtain higher practicality. Methodologically, we propose a generalized and interpretable machine learning approach for football forecasting.

## Materials and methods

In this section, the dataset used and the proposed generalized, interpretable football match result forecast model will be introduced in detail.

### Dataset

Various open-source databases were evaluated in this study, including the European Soccer Database, betting sites, engsoccerdata, and The Open International Soccer Database [30]. We examined the datasets based on the volume of data and the available features. Because the objective of this study is to build a model that is practical to football teams, it is critical to utilize features that directly reflect the team’s quality. Ultimately, the European Soccer Database (https://www.kaggle.com/hugomathien/soccer) from Kaggle was selected as the best database for this study. Although it only contains data from 2008 to 2016, it offers unique features such as team formations, players selected, and the player ratings for each match. These features directly reflect management decisions and the quality of the player. More detail on the dataset is shown in Table 1. Nonetheless, due to a formatting error, we replaced the detailed match statistics with data from the European Soccer Supplementary Database (https://www.kaggle.com/jiezi2004/soccer). For simplicity, we only considered data from the 2011/2012 to 2015/2016 English Premier League (EPL) seasons.

**Table 1:** Dataset features summary.

Dataset Features	Description
Match ID	Unique ID for every match, which acts as the index
Season	Season in which the match was played, e.g. 2011/2012
Round	Competition round of the match, from 1 to 38
Date	Date that the match was played
Match Statistic	Shots on target, Shots off target, Crosses, etc. for both the home and away teams.
Team ID	Unique ID for the home and away team
Team Statistics	Players involved in the match and their formation represented in coordinates
FIFA Rating	38 ratings that reflect each player’s ability/quality
Betting Odds	Betting odds for the match from various providers

### Proposed method

**Fig 1:**
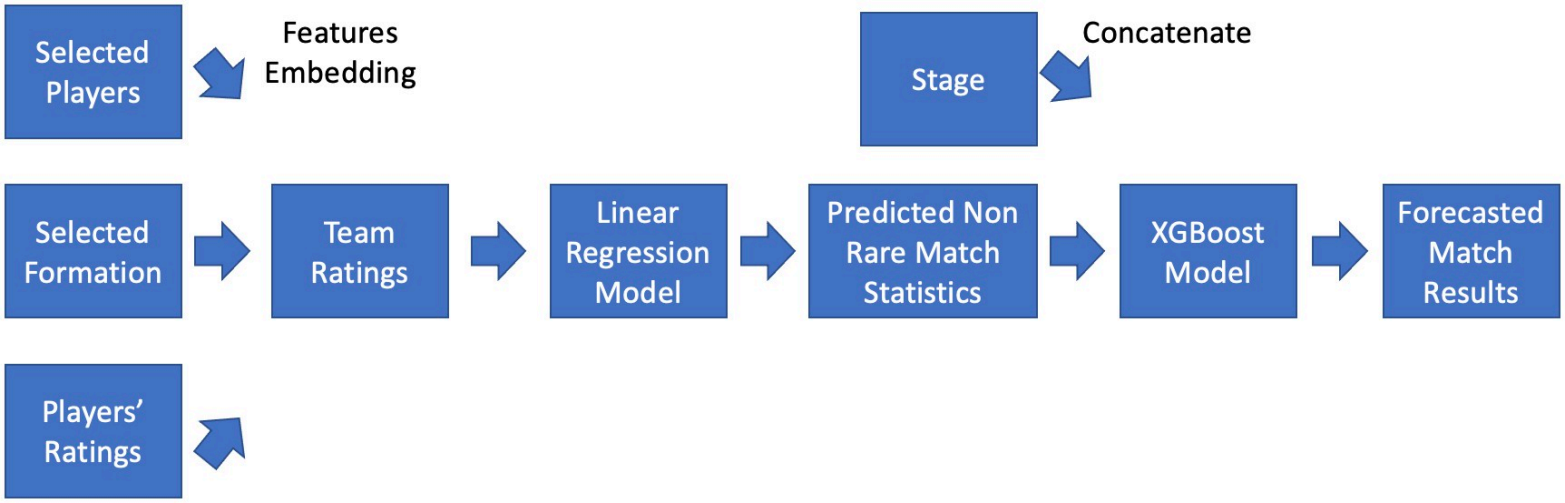
Generalized, interpretable football match result forecasting model architecture.

The generalized, interpretable football match result forecast model consists of four main stages: input feature embedding, non-rare match statistics selection, non-rare match statistics prediction, and match result forecasting. The architecture of the model is shown in Fig 1. Our proposed model was created with the python 3.7.10 packages “scikit-learn” and “xgboost”. We expect our models to be practical to sports participants while having desirable performance.

**Stage 1: Input feature embedding.** Our feature set consists of selected players and formations, and player qualities (FIFA ratings). Using these input features allows sports participants to gain direct control. With the controllable input features, teams can experiment with different combinations to verify their game plan and implement strategies.

In this stage, we aim to reduce the size of the input feature set while retaining the relevant information contained in the features. Before embedding, there were 902 features, in particular, 22 selected player IDs, 22 × 2 player coordinates with respect to length and width—representing the player positions in the team’s formation, and 22 × 38 FIFA ratings that represent the player qualities.

First, we reduced the number of FIFA ratings by assigning the ratings to seven groups, and taking the average of the ratings in each group to create seven new ratings [31]. The details are shown in Table 2. Afterward, since outfield players rarely serve as goalkeepers (and vice versa), the goalkeeping rating was dropped for defenders, midfielders, and attackers, while the skill, attack, and defense ratings were dropped for goalkeepers. This rating grouping method, proposed by [31], was developed in consultation with experts in football, with the aim of producing explainable features. Without the help of an expert in the present study, this method is adopted directly at this stage.

**Table 2:** FIFA ratings grouping assignment [31].

Ratings	Features from”Player_Attributes” Table
Power (POW)	shot_power, jumping, stamina, strength, long_shots
Mentality (MEN)	aggression, interceptions, positioning, vision, penalties
Skill (SKI)	dribbling, curve, free_kick_accuracy, long_passing, ball_control
Movement (MOV)	acceleration, sprint_speed, agility, reactions, balance
Attacking (ATT)	crossing, finishing, heading_accuracy, short_passing, volleys
Defending (DEF)	marking, standing_tackle, sliding_tackle
Goalkeeping (GOL)	gk_diving, gk_handling, gk_kicking, gk_positioning, gk_reflexes

**Fig 2:**
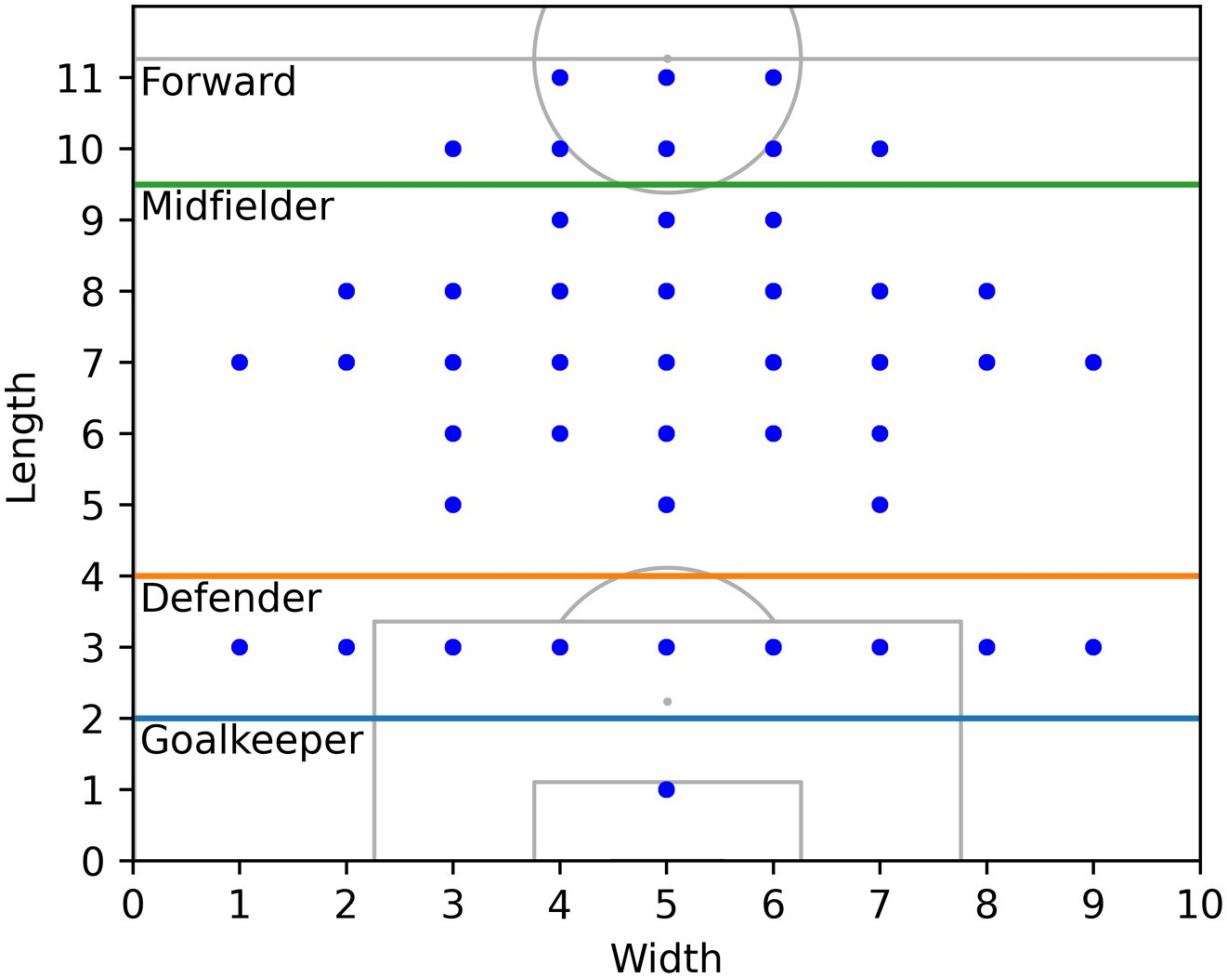
Role assignment based on player coordinates [31].

Second, given the grouped FIFA ratings for each player and the player formation, we further reduced the number of features by grouping the players by role, i.e., forward, midfielder, defender, and goalkeeper according to their coordinates in the formation [31] and aggregated the player ratings in each group. The role assignment procedure is depicted in Fig 2. In the role assignment procedure of [31], each role summarizes each team’s attacking, controlling, defending, and goalkeeping ability.

Unlike [31], we decided to use aggregation of, rather than the averaging of, the player ratings for each role. Averaging ignores the number advantage (e.g., a defensive line comprising five defenders should have greater defensive power than one with four defenders). Further, when the averaging method is applied, the highest rating in a role can be achieved by selecting the one player that has the highest rating. For example, under this method, a defensive line with one high rating defender will outperform a defensive line with four to five players each with slightly low ratings since the former has a higher average rating. This example shows how the averaging method may lead to, and encourage, unrealistic assumptions in terms of formation and player selection. Therefore, to ensure our model can be practical, we replace the averaging method with the aggregation method.

After grouping and aggregating all of the player ratings and player roles, we reduced our number of input features from 902 to 44, while the features are still explainable and practical to sports participants. For clarity and simplicity, we refer to these 44 input features as “team ratings” hereafter.

**Stage 2: Non-rare match statistics selection.** For this stage, we must decide on which non-rare match statistics to utilize, since rare match statistics do not contribute much information to the models [23]. However, rareness is based on subjective judgment. First, from the dataset, corners and throw-ins are combined into crosses as in [23] and other statistics that do not occur frequently: goals, yellow cards, and red cards, are removed. The summary statistics for the remaining match statistics: shots on target, shots off target, shots, and crosses, are presented in Table 3. Since shots on target and shots off target consist of overlapping information with shots, only one of these two features (shots on target and shots off target, or shots) can be selected. Our results show that using shots on target, shots off target, and crosses gives the best performance for the European soccer database data.

**Table 3:** Match statistics’ statistical summary.

Summary Statistics	ShotOn_*h*_	ShotOn_*a*_	ShotOff_*h*_	ShotOff_*a*_	Shots_*h*_	Shots_*a*_	Cross_*h*_	Cross_*a*_
Average (*μ*)	6.6	5.3	6.5	5.1	13.1	10.4	23.6	18.3
Standard Deviation (*σ*)	3.5	3	3.1	2.6	5.3	4.5	8.8	7.5
Minimum (Q0)	0	0	0	0	1	0	2	1
First Quartile (Q1)	4	3	4	3	9	7	17	13
Second Quartile (Q2)	6	5	6	5	13	10	23	18
Third Quartile (Q3)	8	7	8	7	16	13	29	23
Maximum (Q4)	26	19	21	17	39	29	74	56

Table included match statistics: shots on target, shots off target, shots and crosses for the home team (_*h*_) and away team (_*a*_).

**Stage 3: Non-rare match statistics prediction.** In this stage, the input features, the team ratings, from stage 1 are used to predict the non-rare match statistics from stage 2. To perform the prediction, the linear regression model is deployed. This model is selected since it is explainable and has a relatively good performance compared to baseline models. Linear regression is explainable as we can quantify the inference of the input features, the team ratings, on the non-rare match statistics by extracting the standardized regression coefficients (beta terms) from the model.

**Stage 4: Match result forecasting.** In this stage, the predicted non-rare match statistics are first concatenated with the competition round. To forecast match results, the GAP ratings [23] excluded several matches from the beginning and ending competition rounds, which may have differed in terms of team quality and motivation. In this study, we attempted to overcome this limitation by providing more information to the model, specifically the round of the match.

Second, the concatenated features are applied to forecast the football match results. The XGBoost model is selected for forecasting. To improve model performance, model hyperparameters, which are listed below, are tuned with grid search during training. With the trained XGBoost model, the F-score of the model can be extracted to identify the importance of each feature. Further information for model reproduction is given in S1 Appendix and on GitHub (https://github.com/calvinyeungck/Football-Match-Result-Forecast).

- gamma: Minimum gain for further classification tree leaf node splitting. A higher gamma allows for a more generalized XGBoost model.
- learning_rate: Determines how fast the model converges.
- max_depth: The maximum depth of one classification tree in the XGBoost model. For simplicity the maximum is set to 5.
- n_estimator: The number of gradient-boosted classification trees in the XGBoost model. For simplicity, the maximum is set to 300.

**Baseline models.** In this part, baseline models are used to benchmark our proposed method for stage 3 and stage 4. Beginning with Stage 3, non-rare match statistics prediction, we have the following three baseline models.

- Average (AVG): The rolling historical average, a common way for machine learning-based studies to embed the historical match statistics. In this approach, one simply takes the average of the match statistic across previous matches and repeats this averaging when new match data is available.
- GAP rating (GAP): GAP ratings [23], a method to predict non-rare match statistics, has been shown to provide more information in terms of AIC to football match result forecasting models compared to the average [23].
- ANN, LRE: Artificial Neural Network (ANN) and linear regression with elastic net (LRE) are two regression-based models. Both models provide similar performance to the linear regression model we deployed in stage 3. However, the former provides a constant value prediction, which does not provide any information to the XGBoost model we use in stage 4. Also, the latter has the best performance when the hyperparameter alpha and the l1_ratio equal zero, which is identical to our linear regression model. Therefore, these two models will not be discussed further.

For stage 4, match result forecasting, we considered the following three baseline models.

- Betting odds (ODDS): Betting odds-based probabilities have been used in previous studies [27, 32, 33] as baselines for match result forecasting models. By taking the reciprocal of a team’s winning odds and standardizing, the odds can be converted to win probabilities.
- GAP+ Team Rating+ Competition round (GAP+): This model uses a common approach for the machine learning-based model. First, the predicted non-rare match statistics from GAP ratings model, the team ratings, are concatenated with the round. Then, these features are directly input into the XGBoost model.
- Team Rating Only (TR): Using only the team ratings as input features to the XGBoost model. This model aims to verify the necessity of our approach of embedding historical match statistics into the model.

## Results

In this section, the proposed model performance will be verified by comparing it with the corresponding baseline models. Further, the properties of the proposed model in terms of its interpretation and generalization, as well as its stability and practical usage will be discussed.

### Non-rare match statistics prediction performance evaluation

To verify the performance of the proposed model in stage 3 (non-rare match statistics prediction) we selected two baseline models, AVG and GAP. The performance of the models will be evaluated based on their mean absolute error (MAE) and root mean square error (RMSE). The former provides an understanding of general prediction error and the latter penalizes predictions that have large deviations from the target value. The MAE and RMSE of the models for each match statistic are given in S1 and S2 Tables, respectively.

Since each match statistic is on a different scale, it is hard to directly compare model performance across different match statistics with MAE or RMSE. The coefficient of variation (CV) is applied to standardize the error and allows us to compare across match statistics. The CV is obtained by dividing the RMSE by the mean of match statistics (shown in Table 3). The CV of the models for each match statistic are given in Table 4.

**Table 4:** Non-rare match statistics prediction CV.

Match Statistics	AVG	GAP	LR
Home_Shoton	1.10	1.03	1.06
Home_Shotoff	0.92	0.90	0.93
Home_Shots	0.82	0.74	0.79
Home_Cross	0.79	0.75	0.76
Away_Shoton	1.10	1.07	1.09
Away_Shotoff	1.10	1.04	1.04
Away_Shots	0.90	0.82	0.84
Away_Cross	0.85	0.83	0.84

Average (AVG) and GAP rating (GAP) are baseline models. Linear regression (LR) is the non-rare match statistics prediction model in stage 3 of our proposed approach.

From Table 4, comparing across models, GAP had the best performance in predicting all non-rare match statistics, while AVG had the worst performance except for the home team shots off target match statistic. Our linear regression model (LR), in general, had better performance than AVG and matches the performance of GAP for the away team match statistics. In addition, comparing across match statistics, the performance when predicting shots was significantly better than when predicting shots on target and shots off target.

To summarize, GAP had the best performance but the LR of our approach partly matched the performance of GAP, and both methods were better than AVG. This shows that historical match statistics are not the only feasible features to predict future match statistics; more approaches can be considered, typically explainable and practical features as in this study.

### Match result forecasting performance evaluation

We then proceeded to verify the performance of the proposed approach when forecasting match results. For this task, the F1-score and area under the receiver operating characteristic curve (AUC-ROC) were used as the evaluation metrics. The F1-score focuses on precision and recall, while the AUC-ROC focuses on the true positive and false positive rates. These two metrics are commonly applied to evaluate the performance of machine learning models. The forecasting performance in terms of F1-score and AUC-ROC is shown in Table 5.

**Table 5:** Match results forecasting performance.

Scoring rule	GAP+	XGBoost	ODDS	TR
F1-score	0.50	0.47	0.39	0.44
AUC-ROC	0.56	0.54	0.51	0.50
Accuracy	0.58	0.57	0.54	0.50

GAP+ (GAP+ Team Rating+ Competition round), ODDS (Betting odds), and TR (Team Rating Only) are the baseline models. XGBoost is the match results forecasting model in our proposed approach.

From Table 5, GAP+ had the best performance, followed by XGBoost, ODDS, and TR. Further, TR had an AUC-ROC of 0.50, which implies that the TR is likely to be a random classifier. Since the TR model is identical to our proposed model, except for the embedding of the match statistics data into the model. The significant performance advantage of our approach, XGBoost, compare to TR, emphasized the necessity of embedding. Meanwhile, XGBoost had better performance than ODDS, the baseline model commonly applied for football match forecasting studies, showing that our model achieved desirable performance. Even though GAP+ had better performance, unlike our proposed model, it does not provide practical usage.

The main difference between our model and other studies is the features that we used. Since we aim to build a model that could use for tactical decision-making, strength and weakness identification, formation and player selection, and so on. It is important that we select features that are directly related to the team. On the other hand, to the best of our knowledge, recent models achieve F1 scores ranging from 60–70 and they mainly applied features like betting odds, expert opinion, and historical match result-based features. Even though our model might not achieve such performance, our framework is explainable and an AUC and F1 score ranging from 50 to 60 show that information can be derived from the team-related features. We hope that this could inspire future research to develop more team-related features to improve the performance of our framework.

In conclusion, the embedding of historical match statistics was necessary because it allowed our model to have desirable performance and consist of valuable information. Our proposed method traded the performance appropriately, in return for model that is practical to sports participants.

### Match statistics selection

Consider the concept of match statistics prediction. In [23], the concept of GAP is that the predicted non-rare match statistics give less error than AVG, and thus provide more information to the match result forecasting model. Such a concept may lead to an intuition that predicted match statistics with low prediction error will give more information to a match result forecasting model. To verify this intuition, we take a further step to investigate whether the prediction error determines match statistics selection.

In this study, shots on target and shots off target, and shots provide overlapped information. Therefore, for explainability, only one these match statistics can be selected. To determine whether the selection of non-rare match statistics can be based on low prediction error, the error of the match statistics prediction will be discussed first. Afterward, the match result forecasting performance based on different non-rare match statistics selections will be discussed.

As mentioned, the error of shots on target and shots off target was compared to shots. From Table 4, all non-rare match statistics models are able to predict shots with less relative error compared to shots on target or shots off target alone. Additionally, if we consider the match forecasting performance with either shots on target, shots off target, and crosses, or shots and crosses, we can identify that using shots on target and shots off target instead of shots gives better performance. Detailed results are shown in Table 6.

**Table 6:** Models performance with different non-rare match statistics.

Scoring rule	GAP+	GAP+(shots)	XGBoost	XGBoost(shots)
F1-score	0.50	0.49	0.47	0.39
AUC-ROC	0.56	0.55	0.54	0.52

GAP+ (GAP+ Team Rating+ Competition round) and XGBoost utilize non-rare match statistics: shots on target, shots off target, and crosses, where GAP+(shots) and XGBoost(shots) utilize the non-rare match statistics: shots and crosses.

The results imply that selecting non-rare match statistics with low prediction error may sacrifice the information available in the features. Therefore, we have verified that in selecting non-rare match statistics, the prediction error does not indicate which non-rare match statistics to select. Besides, this part filled the gap of GAP ratings [23] in discussing the use of match statistics, shots.

### Model interpretation and generalization

Now that the performance of the model has been verified, the interpretation and generalization of the proposed approach will now be verified.

In stage 3 of our proposed approach, the prediction of non-rare match statistics, the team ratings are used to predict the non-rare match statistics with an LR model. With the standardized regression coefficient (beta term) for each team rating, the effect of each team rating on the non-rare match statistics can be quantified. For example, given the home team’s forward attacking rating, the exact number of home team shots on target, contributed by this rating, can be calculated. Nevertheless, the multicollinearity problem exists in the team ratings, in that there exists a high correlation between ratings, especially between intra-role ratings. Consequently, the standardized regression coefficients may fail to provide a correct indication for the inference of each team rating on the non-rare match statistics.

For stage 4, match result forecasting, the predicted non-rare match statistics and competition rounds are applied to forecast match results with the XGBoost model. The XGBoost model’s f-score, the frequency of a feature being used to split the classification tree, can be extracted to identify the importance of each feature. The f-score of the XGBoost model is given in S1 Fig. Given the multicollinearity problem does not exist, after identifying which non-rare match statistics are important based on the standardized regression coefficients, the important team ratings can be identified.

We now compare our proposed model with common machine learning approaches, for example, GAP+ in model deployment. Our model only requires the user to collect the FIFA ratings of each player, which can be easily collected. No further data collection is required. On the other hand, common machine learning approaches like GAP+ require history-based features or external factors like betting odds and expert opinions being collected. However, to collect such features, match events must already have actually occurred. Therefore, for unmatched teams, teams with debut youth players, newly transferred players, and new formations, the history-based and external features may not be available beforehand. As a result, common machine learning approaches are not able to be deployed. In contrast, FIFA ratings are widely available; even for players in lower divisions and youth players, it allows the proposed model to be generalized, and applicable for almost all combinations of a team, given the FIFA rating is available and disregarded if the match event has already actually occurred.

Despite the current approach not being fully explainable due to the input features, the framework of the proposed approach is fully explainable and is generalized compared to common machine learning approaches. This shows that the proposed approach would be practical and could achieve high performance based on the above results.

### Model stability

In [23], GAP ratings excluded several matches from the first and last six rounds of a season when forecasting match results. Those matches are excluded for model training purposes, and to account for uncertainty in the quality of teams. For example, there may be changes in formations and players at the beginning of the season, and teams might be, in some cases, incentivized to draw in order to secure points at the end of the season.

Therefore, to investigate how the proposed approach performs in these rounds of matches with higher uncertainty, the F1-score and AUC-ROC of the match result forecast, excluding rounds one to six and rounds thirty-three to thirty-eight, is calculated. The exact values of the F1-score and AUC-ROC are reported in S3 Table, and the changes in the F1-score and AUC-ROC are shown in Table 7.

**Table 7:** Match results forecasting models’ performance percentage change after excluding competition rounds 1–6 and 33–38.

	GAP+	XGBoost	ODDS	TR
F1-score	5.70%	2.53%	-1.62%	7.39%
AUC-ROC	4.28%	-0.50%	-0.76%	3.89%

GAP+ (GAP+ Team Rating+ Competition round), ODDS (Betting odds), and TR (Team Rating Only) are baseline models. XGBoost is the match results forecasting model in our proposed approach.

From Table 7, the proposed XGBoost model had the least change in AUC-ROC and second least change in F1-score. This indicates that the model was able to maintain a relatively stable level of performance, even in rounds that have higher uncertainty. Furthermore, GAP+ and TR models were not able to maintain stable performance when predicting the high uncertainty rounds; they are expected to have lower performance. On the other hand, surprisingly, ODDS had a decrease in performance and is expected to perform better in rounds of high uncertainty.

In general, the proposed approach provided stable performance despite the high uncertainty. This would be beneficial when teams have a strategy that they would like to trial, or a potential transfer target, and they would like to try to verify such a highly uncertain team combination with the proposed approach.

### Practical usage of the model

The proposed model can be utilized by a team for strength and weakness identification, formation and player selection, and transfer target validation. The model could even be adopted by lower division teams.

With the F-score of the XGBoost model, teams can understand the relative importance of each non-rare match statistic. Then, with the standardized regression coefficients in the LR model, teams can determine the inference of each of the team ratings to the match results and identify weaknesses or strengths for different roles.

Further, given the FIFA ratings that are available, teams can experiment with different formations and player combinations, with players in the current team and even those from other teams. With the probability forecast function in the XGBoost model, the team can identify their change in win rate based on their hypothesis. Moreover, as FIFA ratings are also available for lower division teams, the proposed model can be adopted by them. Such practicality of the proposed model relies on the fact that the model is interpretable, generalized, stable, and applies explainable and controllable features.

## Discussion

The topic of football match result forecasting has been an ongoing research area with different aims and purposes. This study aimed to propose a practical match result forecasting model for sports participants, which has not been the core focus of prior studies. This study proposed a practical football match result forecasting framework for sports participants. Furthermore, it was shown that the method of embedding historical-based features would be successful.

The practical football match result forecasting framework is a framework that could explain football match results with features that are closely related to the sports participant. Moreover, the framework allows the sports participant to improve, using the information extracted from the framework. Compared to other football match result forecasting frameworks, our framework is able to provide indications of how the coach’s decision and players’ qualities infer the match result. Other frameworks may not be able to provide indications as directly and clearly as ours. However, the practicality comes with a cost in terms of performance, with our framework lagging behind compared to the performance of common machine learning frameworks. Nevertheless, this study has shown the feasibility of creating a practical match result forecasting framework, and future studies can focus on improving the performance of such a framework. Furthermore, this practical framework allows sports participants to extract information from the models, and to verify the extracted information with reality, which could advance the model’s framework in this research domain.

The historical-based features embedding method is a method that allows the model to learn information from historical data. Furthermore, when the model is being applied, it relieved the requirement of retrieving required history, where the history may not be available in advance. The embedding method is based on predicting the historical-based features and subsequently applying the predicted historical-based features. In terms of concept, the method in this study is similar to the concept of GAP ratings [23]. However, the major difference is that [23] predicts the match statistics with historical match statistics. Our model, on the other hand, predicts the match statistics with the coach’s decision and players’ qualities, allowing the framework to be explainable and relieving the requirement of retrieving required history. This allows our framework to be practical. The result of this study has proven that embedding historical-based features is better than discarding the features. This method can be further applied in other machine learning frameworks that may wish to relieve the requirement of retrieving required historical data in model deployment.

Even though our framework is able to achieve high performance and practicality, there are a few limitations to our framework. The first and most significant limitation is the multicollinearity problem in the team ratings. Since this research is conducted without the help of football experts, the method of [31] was applied, with modifications. To resolve the problem, an alternative FIFA ratings grouping method could be applied and other ratings, for example, ratings from football manager game series and football data providers could be considered. Secondly, for simplicity, this study applied only data from the EPL. More football league data could be considered to further evaluate the performance of this framework in other leagues and test its generality. Lastly, the performance of the proposed framework has yet to reach the performance of common machine learning approaches. For simplicity, hyperparameter and model selection in the proposed framework has been limited. The performance of the framework could potentially be improved by replacing the XGBoost model with an XGBoost forest model, and interpretability may be able to be improved with an ADTree model.

This study introduced a practical football match result forecasting framework. In terms of performance, our framework outperformed the universal benchmark, betting odds-based prediction. In terms of interpretation, our model is fully interpretable and practically applicable to sports participants. This model could be utilized by football teams to identify how teams could improve, with a more quantified approach. Moreover, this study has proven the feasibility of, and provide inspiration for, building a practical model in sports.

## Supporting information

S1 Fig F-score for the XGBoost model. The XGBoost model’s f-score, the frequency of a feature being used to split the classification tree, can be used to identify the importance of each feature.
(TIF)

S1 Appendix Generalized interpretable football match result forecasting model reproduction. More information on the model reproduction, including training set and validating set splitting, XGBoost model and hyperparameter tuning, and so on.
(PDF)

S1 Table Non-rare match statistics prediction models performance in terms of MAE. Non-rare match statistics prediction models’ mean absolute error (MAE).
(PDF)

S2 Table Non-rare match statistics prediction models performance in terms of RMSE. Non-rare match statistics prediction models’ root mean square error (RMSE).
(PDF)

S3 Table Match results forecasting models’ performance after excluding competition rounds 1–6 and 33–38. Reporting the exact values of the F1-score and AUC-ROC, to compensate the percentage change information in Table 7.
(PDF)
