# Supplementary figures and images for: A framework of interpretable match results prediction in football with FIFA ratings and team formation
Source: PLoS One. 2023 Apr 13;18(4):e0284318. doi: 10.1371/journal.pone.0284318 (PMC10101499; doi:10.1371/journal.pone.0284318)

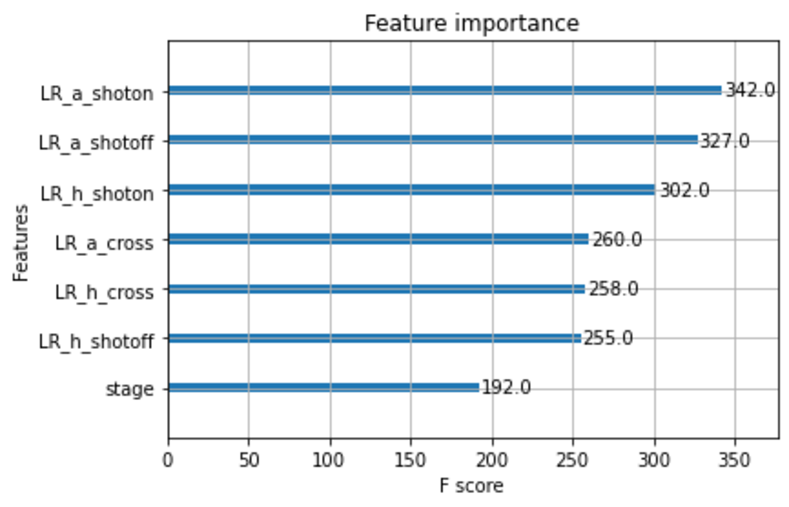

Supplement: S1 Fig — The XGBoost model’s f-score, the frequency of a feature being used to split the classification tree, can be used to identify the importance of each feature. (TIF) [file pone.0284318.s001.tif]
