# Supplementary material for: A framework of interpretable match results prediction in football with FIFA ratings and team formation
Source: PLoS One. 2023 Apr 13;18(4):e0284318. doi: 10.1371/journal.pone.0284318 (PMC10101499; doi:10.1371/journal.pone.0284318)
Supplement: S1 Appendix — More information on the model reproduction, including training set and validating set splitting, XGBoost model and hyperparameter tuning, and so on. (PDF) [file pone.0284318.s002.pdf]

Supplementary Appendix 1

Generalized interpretable football match result forecast model reproduction.

Here, we provide more detail for the reproduction of our proposed model.

Dataset. In the dataset, because of missing data, feature possession and matches with missing player ratings were dropped. The final dataset included 1,784 matches from the original 1,900 matches (comprising 793 wins and 991 draws or losses).

Training set and validation set. To avoid look-ahead bias (using future matches to erroneously predict historical matches) in the training and validation sets. The 2011/2012 and the 2012/2013 seasons were used as the first training set, and the 2013/2014 season was used as the first validation set. The 2012/2013 and 2013/2014 seasons were used as the second training set, and the 2014/2015 season was used as the second validation set to predict future match statistics. The average performance across both validation sets is reported in the results section. For match result forecasting, the predictions on the two validation sets are used as the training set, with the 2015/2016 season serving as the validation set.

Features scaling. There are no much difference in performance with or without min max scaling, the scaling is applied with MinMaxScaler() in sklearn package.

Non rare match statistics prediction. The non-rare match statistics predicition approach in this study is built on the premise that a team with a high attack rating facing a team with a low defense rating is more likely to have more shooting opportunities.

Linear regression model The model is applied with Python, sklearn package, function LinearRegression().

ANN model hyperparameters Grid search shows that ReLu activation function, 1 hidden layer with 10 nodes and a learning rate 0.05 gives the best result.

LRE model hyperparameters Grid search shows that alpha and l1_ratio equal 0 gives the best result.

XGBoost model and hyperparameter tuning. The model is applied with Python, sklearn package, function XGBClassifier(objective="binary:logistic"). To tune the XGBoost model, GridSearchCV() function in sklearn is applied. The function includes cross-validation; however, it has been disabled to avoid look-ahead bias. The tuned hyperparameters' value and the best value for each hyperparameter is reported in the following table.

Table 1. XGBoost model hyperparameters tuning and best results

Hyperparameter	Grid Search Value	Best value
gamma	0,0.1,...,0.5	0
learning_rate	0.1,0.2,...,1	0.1
max_depth	1,2,...,5	3
n_estimator	100,200,300	200

Match result forecast. This task has been considered as a 2-class classification, home team win, and home team draw or lose.
