# Supplementary material for: A framework of interpretable match results prediction in football with FIFA ratings and team formation
Source: PLoS One. 2023 Apr 13;18(4):e0284318. doi: 10.1371/journal.pone.0284318 (PMC10101499; doi:10.1371/journal.pone.0284318)
Supplement: S1 Table — Non-rare match statistics prediction models’ mean absolute error (MAE). (PDF) [file pone.0284318.s003.pdf]

Table 1. Non rare match statistics prediction models performance in MAE

**Match Statistics**	**AVG**	**GAP**	**LR**
Home_Shoton	5.49	**5.30**	5.50
Home_Shotoff	4.71	**4.69**	4.91
Home_Shot	8.21	**7.78**	8.18
Home_Cross	14.13	13.96	**13.85**
Away_Shoton	4.63	**4.38**	4.47
Away_Shotoff	4.61	**4.17**	4.19
Away_Shot	7.71	**6.59**	6.95
Away_Cross	12.60	12.25	**11.98**

Bold result identify the best performance for each match statistic. Average (AVG) and GAP rating (GAP) are the baseline models. Linear regression (LR) is the model in our proposed approach.
