# Supplementary material for: A framework of interpretable match results prediction in football with FIFA ratings and team formation
Source: PLoS One. 2023 Apr 13;18(4):e0284318. doi: 10.1371/journal.pone.0284318 (PMC10101499; doi:10.1371/journal.pone.0284318)
Supplement: S2 Table — Non-rare match statistics prediction models’ root mean square error (RMSE). (PDF) [file pone.0284318.s004.pdf]

Table 1. Non rare match statistics prediction models performance in RMSE

Match Statistics	AVG	GAP	LR
Home_Shoton	7.23	**6.77**	7.02
Home_Shotoff	5.96	**5.88**	6.05
Home_Shots	10.78	**9.75**	10.30
Home_Cross	18.60	**17.78**	17.84
Away_Shoton	5.81	**5.68**	5.75
Away_Shotoff	5.60	5.28	**5.28**
Away_Shots	9.37	**8.48**	8.75
Away_Cross	15.60	**15.25**	15.34

Bold result identify the best performance for each match statistic. Average (AVG) and GAP rating (GAP) are the baseline models. Linear regression (LR) is the model in our proposed approach.
