# Supplementary material for: A framework of interpretable match results prediction in football with FIFA ratings and team formation
Source: PLoS One. 2023 Apr 13;18(4):e0284318. doi: 10.1371/journal.pone.0284318 (PMC10101499; doi:10.1371/journal.pone.0284318)
Supplement: S3 Table — Reporting the exact values of the F1-score and AUC-ROC, to compensate the percentage change information in Table 7. (PDF) [file pone.0284318.s005.pdf]

Table 1. Match results forecasting models' performance after excluding competition rounds 1-6 and 33-38.

Scoring rule	GAP+	XGBoost	ODDS	TR
F1-Score	0.53	0.48	0.38	0.48
AUC-ROC	0.58	0.54	0.51	0.52

GAP+ (GAP+ Team Rating+ Competition round), ODDS (Betting odds), and TR (Team Rating Only) are the baseline models. XGBoost is the match results forecasting model in our proposed approach.
